# Expression and Function of Granzymes A and B in *Escherichia coli* Peritonitis and Sepsis

**DOI:** 10.1155/2017/4137563

**Published:** 2017-06-12

**Authors:** M. Isabel García-Laorden, Ingrid Stroo, Sanne Terpstra, Sandrine Florquin, Jan Paul Medema, Cornelis van´t Veer, Alex F. de Vos, Tom van der Poll

**Affiliations:** ^1^Center for Experimental and Molecular Medicine (CEMM), Academic Medical Center, University of Amsterdam, Meibergdreef 9, 1105 AZ Amsterdam, Netherlands; ^2^Department of Immunopathology, Sanquin Research, Plesmanlaan 125, 1066 CX Amsterdam, Netherlands; ^3^Department of Pathology, Academic Medical Center, University of Amsterdam, Meibergdreef 9, 1105 AZ Amsterdam, Netherlands; ^4^Laboratory for Experimental Oncology and Radiobiology (LEXOR), Academic Medical Center, Meibergdreef 9, 1105 AZ Amsterdam, Netherlands; ^5^Division of Infectious Diseases, Academic Medical Center, University of Amsterdam, Meibergdreef 9, 1105 AZ Amsterdam, Netherlands

## Abstract

*Escherichia* (*E*.) *coli* is the most common causative pathogen in peritonitis, the second most common cause of sepsis. Granzymes (gzms) are serine proteases traditionally implicated in cytotoxicity and, more recently, in the inflammatory response. We here sought to investigate the role of gzms in the host response to *E. coli*-induced peritonitis and sepsis in vivo. For this purpose, we used a murine model of *E. coli* intraperitoneal infection, resembling the clinical condition commonly associated with septic peritonitis by this bacterium, in wild-type and gzmA-deficient (*gzmA^−/−^*), *gzmB^−/−^*, and *gzmAxB^−/−^*mice. GzmA and gzmB were predominantly expressed by natural killer cells, and during abdominal sepsis, the percentage of these cells expressing gzms in peritoneal lavage fluid decreased, while the amount of expression in the gzm^+^ cells increased. Deficiency of gzmA and/or gzmB was associated with increased bacterial loads, especially in the case of gzmB at the primary site of infection at late stage sepsis. While gzm deficiency did not impact neutrophil recruitment into the abdominal cavity, it was accompanied by enhanced nucleosome release at the primary site of infection, earlier hepatic necrosis, and more renal dysfunction. These results suggest that gzms influence bacterial growth and the host inflammatory response during abdominal sepsis caused by *E. coli*.

## 1. Introduction

Sepsis is the leading cause of death in critically ill patients in developed countries. Peritonitis, infection of the intra-abdominal cavity, is the second most common cause of sepsis [1], with *Escherichia* (*E*.) *coli* being the pathogen most commonly involved [2]. Abdominal sepsis bears a grim prognosis with mortality rates up to 60% when accompanied by shock [3]. While an immediate and adequate immune response is necessary to contain and kill the pathogen, aberrant immune activation can contribute to collateral damage and tissue injury [4].

Granzymes (gzms) are a family of serine proteases. While mice express gzms A–G, K, M, and N, humans only have five different gzms (A, B, H, K, and M) [5]. The most abundant gzms, gzmA and gzmB, are constitutively expressed in several cell types including cytotoxic T lymphocytes (CTL), natural killer (NK) cells, NKT cells, and *γδ* T cells [6, 7]; their expression has been also observed in other cell types, including nonlymphoid cells, at least after stimulation [8, 9]. A role of gzms in eliminating infected, neoplastic, or foreign cells has been described in numerous studies, but the physiological relevance of gzmA cytotoxicity is still controversial [5]. Both gzmA and gzmB plasma levels have been found elevated in patients with diverse parasitic, viral, and bacterial infections [8, 10] and with severe sepsis [11, 12], as well as in healthy individuals with experimentally induced endotoxemia [13]. Induction of gzmA and gzmB secretion has also been reported after stimulation of whole blood with gram-negative and gram-positive bacteria [13]. Moreover, a role for gzmA and gzmB in mediating cytokine release or maturation has been documented [14]. Of interest, gzmA- and gzmB-deficient mice have been shown to be relatively protected against endotoxin-induced shock [15, 16]. Altogether, these studies point to a role for gzms in infection and the accompanying inflammatory response that extends beyond gzm-mediated cytotoxicity.

Current knowledge on the role of gzms in the host response to *E. coli* and in the pathogenesis of peritonitis and sepsis is highly limited. In the present study, we aimed to investigate the role of gzmA and gzmB in the host response to *E. coli*-induced peritonitis and sepsis using a murine model, analyzing the local and systemic inflammatory response and damage in wild-type (WT) mice and mice deficient in gzmA, gzmB, or both gzms.

## 2. Materials and Methods

### 2.1. Animals

C57BL/6 WT mice were purchased from Charles River Laboratories Inc. (Maastricht, Netherlands). GzmA-deficient (*gzmA^−/−^*), *gzmB^−/−^*, and *gzmAxB^−/−^* mice on a C57BL/6 background were kindly provided by Dr. M. M. Simon (Max Planck Institute, Freiburg, Germany) [17–19]. These genetically modified mice have shown to have normal immune cell profiles at baseline [17, 18]. All experiments were conducted with mice between 10 and 12 weeks of age. Experimental groups, consisting of both male and female mice, were age- and gender-matched and housed in the Animal Research Institute Amsterdam under pathogen-free conditions, receiving food and water ad libitum, with a 12-hour day-night rhythm, and daily checks. Upon arrival in the facility, mice were acclimatized for at least 7 days before use in experiments. All experiments were carried out in accordance with the Dutch Experiment on Animals Act and were approved by the Animal Care and Use Committee of the Academic Medical Centre (Permit numbers DIX32AA and DIX32AB). Mice were sacrificed by cervical dislocation under 5% isoflurane anesthesia, and all efforts were made to minimize suffering.

### 2.2. Experimental Peritonitis and Sample Collection

Peritonitis was induced by intraperitoneal inoculation with 1.3∗10^4^ colony-forming units (CFU) of *E. coli* serotype O18:K1 in 200 *μ*l saline solution as previously described [20]. The bacterial dose was based on our previous studies and known to cause severe disease associated with rapid growth of the pathogen and dissemination to distant body sites [20, 21]. Mice were euthanized at 6, 14, or 20 hours after infection (*N* = 8 mice per group at each time point), and samples were collected and processed as described [20]. Briefly, peritoneal lavage fluid (PLF) was collected after peritoneal lavage with 5 ml of sterile PBS, blood was drawn into heparinized tubes, and lung and liver were harvested. The right lung and a piece of liver were weighed and diluted 1 : 4 in sterile isotonic saline and homogenized using a tissue homogenizer. To determine bacterial loads, the blood, PLF, and tenfold dilutions of the homogenates were plated on blood agar plates, and colonies were counted after incubation at 37°C for 16 h. For histology, the left lung and a piece of liver were fixed in 10% buffered formalin and embedded in paraffin. Total cell counts were quantified from PLF using a hemocytometer. Subsequently, PLF was centrifuged at 1400 ×g for 10 min, and the supernatant was collected in sterile tubes and stored at −20°C until determination of cytokines, while the pellet was used to perform differential cell counts on Giemsa-stained cytospin preparations (Diff-Quick) by direct counting on a microscope. To analyze the basal levels of gzmA and gzmB, groups of WT mice were euthanized before and after infection with *E. coli*, and the PLF, blood, lung, and liver were harvested and processed as described to determine bacterial loads (*N* = 11) and/or processed for flow cytometry (*N* = 6).

### 2.3. Histopathology

Four micrometer organ sections were stained with hematoxylin and eosin and analyzed using a semiquantitative scoring system by a pathologist who was blinded for groups [20]. Liver injury was scored according to the parameters interstitial inflammation (on a scale from 0—condition absent to 4—very severe), number of thrombi, and percentage of hepatocellular necrosis. To score lung inflammation and damage, the parameters interstitial inflammation, endothelialitis, edema, pleuritis, and presence of thrombi were used. Each parameter was rated separately on a scale from 0 to 4 (as for liver). The total lung inflammation score was expressed as the sum of the scores of the individual parameters, the maximum being 20. Images were acquired with a camera under light microscopy at 10x.

### 2.4. Assays

PLF and plasma levels of interleukin- (IL-) 10, tumor necrosis factor- (TNF-) *α*, interferon- (IFN-) *γ*, and monocyte chemotactic protein-1 (MCP-1) were measured by cytometric bead array multiplex assay (BD Biosciences, San José, CA, USA) according to the manufacturer's recommendations. IL-1*β*, IL-6, and cytokine-induced neutrophil chemoattractant (KC, CXCL1) were measured by ELISA (R&D Systems, Abingdon, UK) in accordance with the manufacturer's instructions. Lactate dehydrogenase (LDH), aspartate aminotransferase (ASAT), alanine transaminase (ALAT), creatinine, and urea levels were measured in plasma using kits from Sigma (St. Louis, MO, USA) by a Hitachi analyzer. Nucleosome levels were determined in PLF by ELISA as described previously [22].

### 2.5. Flow Cytometry

Flow cytometry was done as described [23]. In brief, blood erythrocytes were lysed with ice-cold isotonic NH_4_Cl solution, and the remaining cells were washed and counted by using a hemocytometer. As for PLF, counted cells were resuspended in FACS buffer (PBS supplemented with 0.5% BSA, 0.01% NaN3, and 0.35 mM EDTA). Immunostaining for cell surface molecules was performed for 25 min at 4°C in the dark, using the following antibodies: PerCP-Cy5.5 rat anti-mouse CD3 (clone 17A2; BD Pharmingen, San Jose, CA, USA), AF700 rat anti-mouse CD4 (clone RM4-5; BD Pharmingen), APC-Cy7 rat-anti-mouse CD8 (clone 53-6.7; Biolegend, San Diego, CA, USA), APC hamster anti-mouse *γδ* TCR (clone GL3; eBioscience, San Diego, CA, USA), and PE-Cy7 mouse-anti-mouse NK1.1 (clone PK136; eBioscience). For the intracellular staining, cells were fixed and resuspended in perm/wash buffer containing the antibodies PE mouse anti-mouse granzyme A (clone 3G8.5; Santa Cruz Biotechnology, Dallas, TX, USA) and PE-CF594 mouse anti-human granzyme B (clone GB11; BD Horizon, San José, CA, USA). This clone of anti-human gzmB antibody has been shown to recognize mouse gzmB [24]. All antibodies were used in concentrations recommended by the manufacturer. The cells were analyzed by flow cytometry with a FACSCanto (BD Bioscience). The FlowJo (Tree Star Inc., Ashland, OR, USA) and BD FACSDiva (BD Bioscience) softwares were used to analyze the data. Based on the forward scatter versus side scatter dot plot, the area containing the leukocytes was gated. Cells were selected as CD3^+^CD4^+^ (CD4^+^ T), CD3^+^CD8^+^ (CD8^+^ T), CD3^+^*γδ*TCR^+^ (*γδ* T), CD3^+^NK1.1^+^ (NK1.1^+^ T), and CD3^−^NK1.1^+^ (NK), and expression of gzmA and gzmB was analyzed in these populations. The results are expressed as percentage of cells of the specific lymphocyte population expressing the corresponding gzm and as the median fluorescence intensity of the expression (MFI). Alternatively, cells were selected as positive for each gzm and the percentages of the abovementioned lymphocyte populations were analyzed within the gzm^+^ live cells.

### 2.6. Statistical Analysis

Data are expressed as box-and-whisker diagrams showing the smallest observation, lower quartile, median, upper quartile and largest observation, or as medians with interquartile ranges. Comparisons between multiple groups were performed using the Kruskall-Wallis test and between two independent groups using Mann–Whitney *U* test. Analyses were done using GraphPad Prism version 4.0 and SPSS Statistical Package version 15.0. Statistical significance was set at *P* value <0.05.

## 3. Results

### 3.1. Expression of Granzymes A and B during *E. coli* Peritonitis

**Figure 1:**
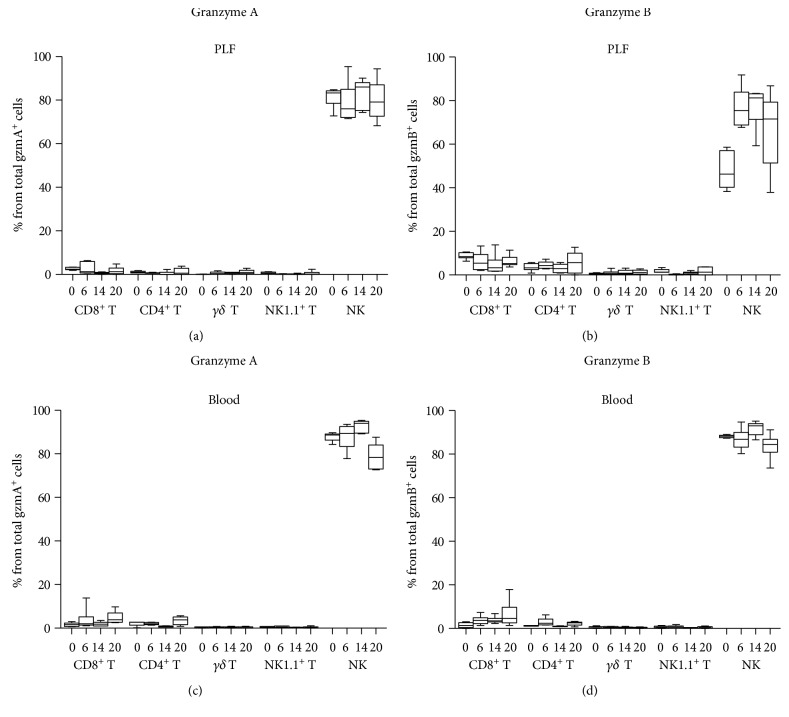
Lymphocyte source of intracellular granzymes A and B in peritoneal lavage fluid (PLF) and blood of wild-type mice before and after intraperitoneal infection with *E. coli*. Mice were infected intraperitoneally with 1.3∗10^4^ CFU *E. coli* and sacrificed before and at 6, 14, and 20 h after infection. To show the lymphocyte source of each granzyme, the total gzmA- or gzmB-positive cells were selected and, the lymphocyte subsets to which those gzm-expressing cells belong, identified. (a) and (c) show the percentage of the total gzmA and (b) and (d) of gzmB expressed by different lymphocyte subsets from PLF ((a) and (b)) and blood ((c) and (d)) are shown. Data are box-and-whisker diagrams depicting the smallest observation, lower quartile, median, upper quartile, and largest observation. *N* = 5-6 at each time point.

To gain insight into the role of gzmA and gzmB in peritonitis and sepsis, we first sought to ascertain the expression of these gzms before and during *E. coli* peritonitis. For this purpose, we infected WT mice intraperitoneally and analyzed the intracellular expression of gzmA and gzmB before and 6, 14, and 20 h after infection by flow cytometry. Only a low proportion of live cells from the blood (<6%) and even lower from PLF (<1.5%) expressed gzms (data not shown). The most relevant lymphocyte subpopulations previously implicated in gzm expression (NK, NK1.1^+^ T, *γδ* T, CD8^+^ T, and CD4^+^ T cells) [8] were studied (the gating strategy for each subpopulation is shown in Supplementary Figure 1A available online at https://doi.org/10.1155/2017/4137563). As expected [23], NK cells were by far the main cellular source of gzmA and gzmB in both PLF and blood at every time point (Figure 1(); gating strategy shown in Supplementary Figure 1B).

**Figure 2:**
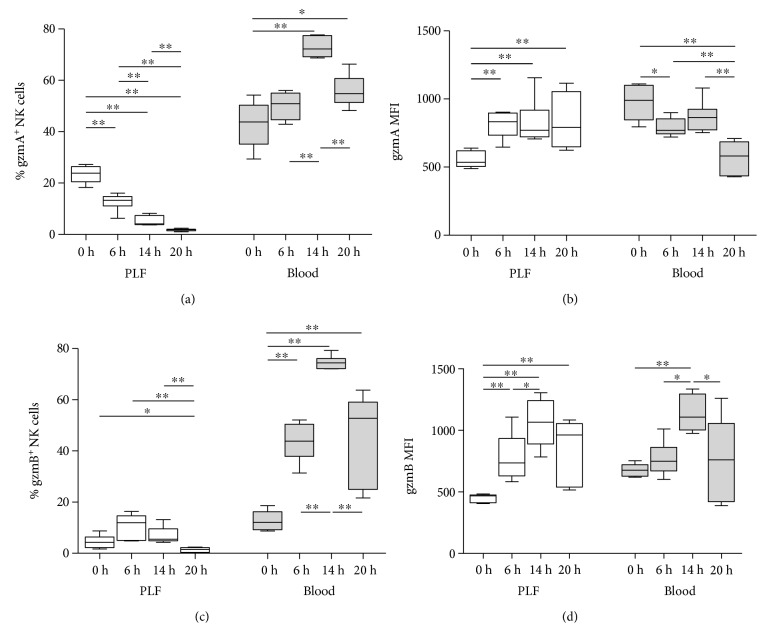
Granzyme A and B expression in NK cells from wild-type mice before and after intraperitoneal infection with *E. coli*. Mice were infected intraperitoneally with 1.3∗10^4^ CFU *E. coli* and sacrificed before and at 6, 14, and 20 h after infection. Percentage and median fluorescence intensity (MFI) of intracellular gzmA (a, b) and gzmB (c, d) in NK cells from peritoneal lavage fluid (PLF) and blood are shown. Data are box-and-whisker diagrams depicting the smallest observation, lower quartile, median, upper quartile, and largest observation. *N* = 5-6 at each time point. ^∗^*P* < 0.05, ^∗∗^*P* < 0.01 determined by Mann–Whitney *U* test.

We then analyzed the percentage of cells that were gzm^+^ within each lymphocyte population, as well as the MFI of these cells (Figure 2 and Supplementary Table 1; gating strategy shown in Supplementary Figure 1A). In PLF, the percentage of NK cells expressing gzmA clearly decreased over time, while the MFI (i.e., the amount of gzmA expressed by gzmA^+^ NK cells) increased after infection (Figures 2(a) and 2(b)). In contrast, the percentage of CD3^+^ cells (CD8^+^ T, CD4^+^ T, *γδ* T, and NK1.1^+^ T cells) that were gzmA^+^ increased during peritonitis (Supplementary Table 1). In blood, a higher percentage of NK cells expressed gzmA when compared to PLF NK cells, peaking at 14 h (Figure 2(a)), while the MFI decreased after infection, especially at 20 h (Figure 2(b)). GzmB increased in NK cells after induction of peritonitis in both PLF and blood, expressed either as percentage of positive cells and MFI (Figures 2(c) and 2(d)). Cell numbers and percentage of NK cells in PLF, as well as numbers of NK cells that were gzm^+^, are presented in Supplementary Table 2, showing that there is an absolute and relative increase in NK cells over time and that the decrease in the percentage of gzmA^+^ NK cells is not accompanied by a decrease in the numbers of these cells.

### 3.2. Effect of Granzyme A and B Deficiency on Bacterial Outgrowth during *E. coli* Peritonitis

**Figure 3:**
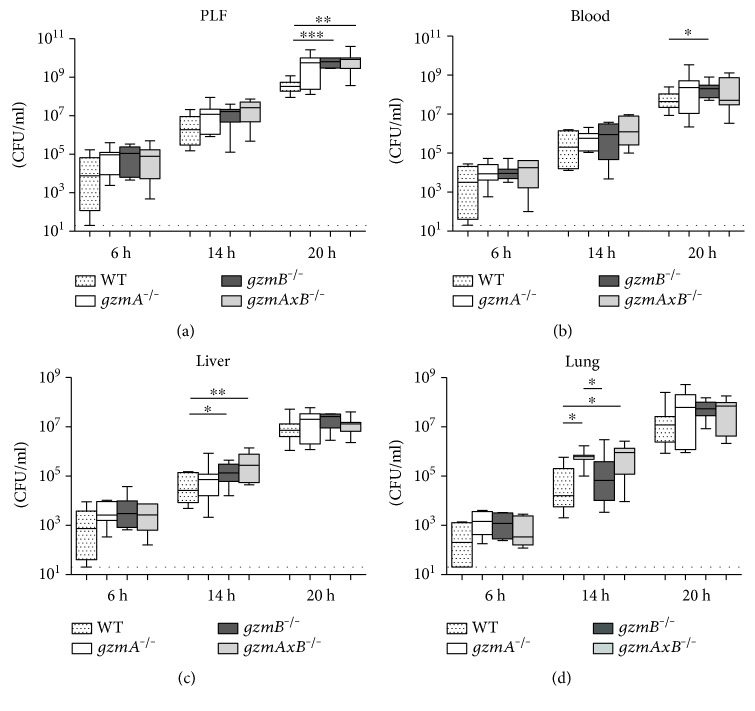
Bacterial loads in peritoneal lavage fluid (PLF), blood, liver, and lung of wild-type, *gzmA^−/−^*, *gzmB^−/−^*, and *gzmAxB^−/−^* mice during *E. coli* peritonitis. Mice were infected intraperitoneally with 1.3∗10^4^ CFU *E. coli* and sacrificed at 6, 14, and 20 h after infection. Data are box-and-whisker diagrams depicting the smallest observation, lower quartile, median, upper quartile, and largest observation. *N* = 7-8 per group at each time point. ^∗^*P* < 0.05, ^∗∗^*P* < 0.01, and ^∗∗∗^*P* < 0.001 by Mann–Whitney *U* test. Dotted line represents the lower detection limit of CFU.

With the aim of evaluating whether gzmA and/or gzmB deficiency influences local bacterial outgrowth and subsequent dissemination, we determined bacterial loads in the PLF, blood, and liver and lung homogenates of WT, *gzmA^−/−^*, *gzmB^−/−^*, and *gzmAxB^−/−^* mice at predefined time points after infection (Figure 3). At the early time point (6 h), the infection had already disseminated, but no significant differences were observed between groups at any body site. At 14 h, gzm knockout (KO) mice presented higher CFUs than WT mice in distant organs; in particular, in the lung, *gzmA^−/−^* showed increased bacterial loads compared to WT and *gzmB^−/−^* mice, and *gzmAxB^−/−^* compared to WT mice, while in the liver, *gzmB^−/−^* and, more significantly, *gzmAxB^−/−^* presented more CFUs than WT mice. At 20 h, *gzmB^−/−^* mice in blood and, more remarkable, *gzmB^−/−^* and *gzmAxB^−/−^* mice in PLF had higher bacterial loads relative to WT mice.

### 3.3. Role of Granzyme A and B Deficiency in the Inflammatory Response during *E. coli* Peritonitis

**Figure 4:**
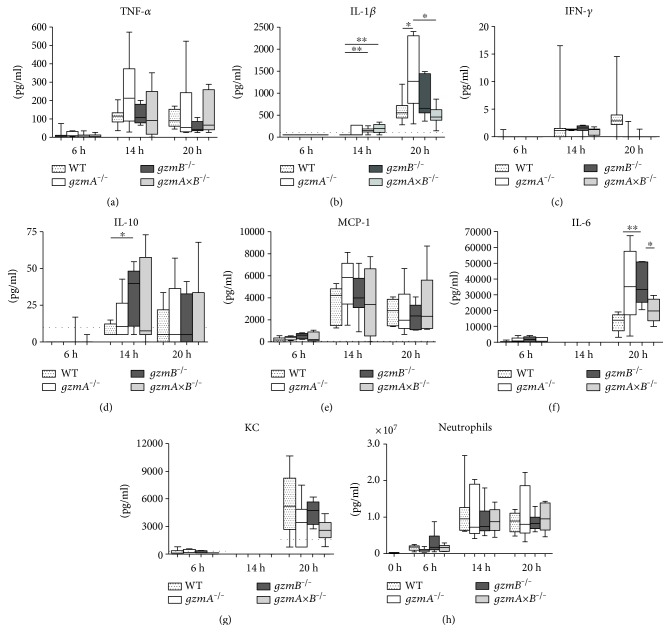
Cytokine and chemokine levels as well as neutrophil counts in peritoneal lavage fluid (PLF) of wild-type, *gzmA^−/−^*, *gzmB^−/−^*, and *gzmAxB^−/−^* mice during *E. coli* peritonitis. Mice were infected intraperitoneally with 1.3∗10^4^ CFU *E. coli* and sacrificed at 6, 14, and 20 h after infection. Data are box-and-whisker diagrams depicting the smallest observation, lower quartile, median, upper quartile, and largest observation. *N* = 7-8 per group at each time point. ^∗^*P* < 0.05 and ^∗∗^*P* < 0.01 determined by Mann–Whitney *U* test. Dotted lines represent lower detection limits. NA: not available.

Cytokines are essential mediators of the host response to infection. To get insight into the influence of gzmA and gzmB deficiency on the local inflammatory response elicited during septic peritonitis, we measured proinflammatory (TNF-*α*, IFN-*γ*, and IL-1*β*, IL-6) and anti-inflammatory (IL-10) cytokines as well as MCP-1 and KC levels in PLF. Six hours after inoculation, the levels were low and did not differ between the four groups of mice (Figure 4). At later time points during the infection, cytokine levels in PLF showed strong interindividual variation with some differences between mouse strains. At the intermediate time point (14 h), relative to WT mice, *gzmB^−/−^* mice had higher PLF IL-1*β* and IL-10 levels, whereas *gzmAxB^−/−^* mice displayed higher levels of IL-1*β* (Figures 4(b) and 4(d)). During late stage peritonitis (20 h), *gzmA^−/−^* mice presented with the highest PLF IL-1*β* levels (Figure 4(b)), and *gzmB^−/−^* mice showed higher levels of IL-6 than WT mice (Figure 4(f)). We also determined the plasma concentrations of cytokines and MCP-1 as a readout for systemic inflammation; although some modest differences were found between groups, there was no consistent pattern identifying a clear role for gzmA or gzmB (Supplementary Table 3).

In addition, we assessed cell recruitment into the site of infection by counting the number of neutrophils in PLF of the 4 groups of mice at the different time points. Peritonitis was associated with an influx of neutrophils into PLF in all groups without differences between strains (Figure 4(h)).

### 3.4. Effect of Granzyme A and B Deficiency on Organ Injury during *E. coli* Peritonitis

**Figure 5:**
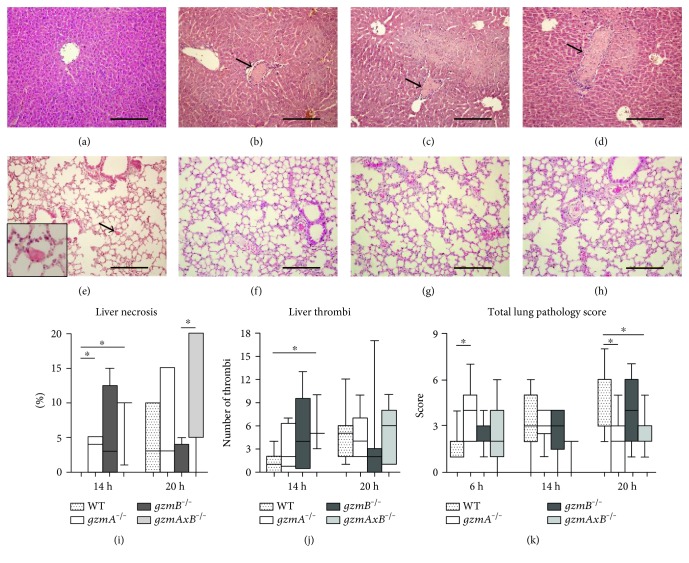
Histopathology of the liver and lung from wild-type, *gzmA^−/−^*, *gzmB^−/−^*, and *gzmAxB^−/−^* mice during *E. coli* peritonitis. Mice were infected intraperitoneally with 1.3∗10^4^ CFU *E. coli* and sacrificed at 6, 14, and 20 h after infection. Pictures of hematoxylin-eosin staining of representative tissue samples are shown; original magnification 10x; inset shows a 40x magnification of a thrombus (arrow); scale bars: 200 *μ*m. For each organ slide (1 per mouse), the whole section was examined and scored. (a), (b), (c), and (d) show, respectively, wild-type and *gmzA^−/−^, gzmB^−/−^*, and *gzmAxB^−/−^* mice ((b), (c), and (d) presenting necrotic areas) livers 14 h after infection. Necrosis (expressed as percentage in (i)) and thrombi (j) were found at 14 and 20 h in the liver. (e), (f), (g), and (h) show, respectively, wild-type and *gmzA^−/−^, gzmB^−/−^*, and *gzmAxB^−/−^* mice lungs 20 h after infection. Semiquantitative histology total score of lung slides are represented (k). Data are box-and-whisker diagrams depicting the smallest observation, lower quartile, median, upper quartile, and largest observation. *N* = 7-8 per group at each time point. ^∗^*P* < 0.05 determined by Mann–Whitney *U* test.

Considering the quick and widespread dissemination of the pathogen, we investigated the effect of gzmA and gzmB deficiency in distant organs during the progression of the disease. For this purpose, we semiquantitatively analyzed liver and lung histology slides prepared from *gzmA^−/−^*, *gzmB^−/−^*, and *gzmAxB^−/−^* mice 6, 14, and 20 h after infection. At 6 h, we observed some hepatic inflammation, which was significantly more pronounced in *gzmA^−/−^* than in WT mice (Supplementary Figure 2). Remarkably, at 14 h, all gzm-deficient mouse strains but not WT mice showed signs of liver necrosis (Figures 5(a), 5(b), 5(c), 5(d), and 5(i)), which was accompanied by higher numbers of thrombi, significantly so in *gzmAxB^−/−^* mice (Figure 5(j)). In lungs, pathology scores remained low in all groups, with *gzmA^−/−^* and *gzmAxB^−/−^* mice showing lower total pathology score at 20 h when compared with WT mice, which was caused by a reduced number of thrombi in the lungs (Figures 5(e), 5(f), 5(g), 5(h), and 5(k), and Supplementary Figure 2).

To obtain further insight into distant organ injury at late stage peritonitis, we analyzed the plasma concentrations of ASAT and ALAT (both parameters of hepatocellular injury), creatinine and urea (for renal function), and LDH (indicative for cellular injury in general) at 20 h (Table 1). Gzm-deficient mice showed higher plasma levels of creatinine than WT mice. Finally, we measured PLF levels of nucleosomes as a marker of cell death at the primary site of infection. At early and middle time points, the nucleosome levels were undetectable for all the mice strains. However, at 20 h, there was a significant increase in the three gzm KO groups when compared with WT mice (Table 1).

## 4. Discussion

Peritonitis can rapidly turn into life-threatening sepsis. In this intra-abdominal infection, *E. coli* is the most commonly found pathogen [2]. Given that previous studies have reported elevated levels of gzmA and gzmB in plasma of patients with severe sepsis [11, 12], and that gram-negative bacteria, as well as *E. coli* lipopolysaccharide (LPS), are potent inducers of in vitro release of both gzms [13], we sought to investigate the role of these gzms in the host response to *E. coli*-induced peritonitis and sepsis in vivo. For this purpose, we used a murine model of *E. coli* abdominal infection in WT mice and mice deficient in gzmA and/or gzmB. Our study showed an association of gzm deficiency with enhanced bacterial growth, as well as an association with differences in organ damage in advanced peritonitis and sepsis.

The present study is the first, to our knowledge, showing the pattern of intracellular expression of gzmA and gzmB by different lymphocyte populations before and after induction of *E. coli* infection. As expected [23], NK cells were the main cell type expressing gzms. The percentage of NK cells in PLF-expressing gzmA decreased over time; a similar pattern was seen for gzmB, albeit to a lesser extent and after an initial not significant increase. This could be largely due to the occurrence of new (still gzm negative) NK cells, since NK cell numbers in PLF increased after infection. Indeed, the level of intracellular expression in gzm-positive cells increased in time. This differs from our previous findings in a murine model of pneumonia [23], where the proportion of gzmA^+^ NK cells at the primary site of infection (the lungs) remained stable until late pneumosepsis, when there was a strong fall, while the MFI decreased in time. This points towards differences in the response depending on the pathogen and the primary site of infection. Curiously, in blood, the percentage of NK cells expressing gzmA and gzmB increased after *E. coli* infection, suggestive of differential responses at the primary site of infection and systemically. Unfortunately, we could not assess the impact of these observations on extracellular levels of gzms during *E. coli* infection, due to the lack of mouse specific immune assays with sufficient sensitivity.

Next, we analyzed the effect of deficiency of gzms A and B on the host response during *E. coli* peritonitis and sepsis. For this purpose, we used an established model of sepsis by *E. coli* [20, 21], which is the most common causative agent in sepsis due to peritonitis. In this model, the host immune system is challenged with a virulent strain of *E. coli* that rapidly grows and disseminates, producing an early systemic inflammatory response, and mimicking the condition of severe abdominal sepsis [20, 21]. Notably, the predefined endpoints to study host response parameters were based on a previous work from our group, providing insight in both the primary (6 h) and late (20 h) responses, the latter time point being just before the first deaths are expected [20, 21]. As such, this model is suitable to study both the early response at the primary site of infection and the late local and systemic damage. To our knowledge, there are no studies of abdominal sepsis in gzm-deficient mice using this or a different *E. coli* strain. The use of an only strain is a limitation of our study, and investigations using other *E. coli* strains would be interesting in order to confirm our results. First of all, we observed increased bacterial loads in distant organs of gzm-deficient mice at intermediate time point and, more significantly, in PLF at late time point, indicating a role of the gzms, mainly gzmB, in controlling bacterial outgrowth. A previous study reported a delay in bacterial clearance in the spleen, but not in the liver, of *gzmB^−/−^* and *gzmAxB^−/−^* compared to WT mice infected intraperitoneally with the intracellular gram-negative bacterium *Brucella microti* [25]. Our previous study on the role of gzms during *Klebsiella pneumoniae* pneumonia, also a gram-negative pathogen, showed temporarily increased bacterial loads in the lungs but not in distant organs in *gzmA^−/−^* and *gzmAxB^−/−^* mice [23], suggesting that the role of gzms in antibacterial defense varies depending on the pathogen and the site of the original infection.

Early after infection (6 h), all mouse strains presented similar organ pathology and low PLF cytokine levels. A limitation of our study is that we did not obtain insight into alterations in Th17 cells or IL-17 levels, which are major players in protective immunity during bacterial infection [26]. At the intermediate time point (14 h), gzm KO mouse strains (mainly *gzmAxB^−/−^*), but not WT mice, presented necrosis and higher numbers of thrombi in the liver. This could be reflecting, at least for the double KO mice, the increased bacterial loads found in the liver at this time point. However, the differences in bacterial burdens observed in the lung were not reflected in lung pathology. The strongest differences in bacterial loads (WT versus *gzmB^−/−^* and *gzmAxB^−/−^* mice) were found in PLF at late peritonitis (20 h), but these differences were neither reflected in the liver and lung damage or cytokine levels. Taking all this into account, it does not seem that the observed phenotype of inflammation and damage is caused by the differences in containment of bacterial growth. Granzyme deficiency differentially influenced pathology in the lungs and liver. Our study does not provide insight in the underlying mechanism of this finding; possible explanations include differential influx of gzm-expressing cells and gzm release in different organs. The lungs of gzm-deficient mice contained less thrombi after induction of peritonitis, which likely is beneficial to the host, considering that microvascular thrombosis in the lung vasculature may contribute to lung injury during sepsis from an extrapulmonary source [27]. Granzyme-deficient mice showed earlier liver damage and more renal damage and local cell death when compared with WT mice, as indicated by an early occurrence of liver necrosis, an increase in creatinine levels and presence of PLF nucleosomes in the KO mice. In earlier studies, we also documented distant organ injury in this model in various mouse strains [28, 29]. While multiple organ failure is a common complication in abdominal sepsis [3, 4], a possible protective role of gzms herein in human sepsis remains to be established. Notably, *gzmB^−/−^* mice showed lower sepsis scores than WT mice in a model of polymicrobial peritonitis induced by cecal ligation and puncture [30]. Clearly, this model is different from the model used here, involving local abscess formation, a necrotic part of the bowel, and a more acute disease course, which at least in part can explain the seemingly contradictory results. In addition, taking into account the findings related to bacterial burdens and organ damage, and considering that gzms have been previously implicated in the production, release, and/or processing of proinflammatory cytokines [9, 14], it is remarkable that gzm deficiency had little impact on cytokine production in our study. There is a possibility that deficiency of granzymes affects the ontogeny of the immune system, which could influence our results. However, the KO mice strains used in this study have shown to have normal immune cell profiles at baseline [17, 18].

Most knowledge of the biology of gzms is derived from studies on apoptosis of malignant or virus-infected cells [8, 14]. In the present study, we report the intracellular expression of gzmA and gzmB and their function in a mouse model that resembles the clinical condition commonly associated with septic peritonitis by *E. coli*. NK cells were shown to be the predominant cell-expressing gzms, and deficiency of gzmA and gzmB was associated with increased bacterial loads accompanied by augmented nucleosome release at the primary site of infection (suggestive of local cell death), earlier signs of liver necrosis, and more renal dysfunction. While these results provide insight into the role of gzms in the host response during abdominal sepsis, further research is warranted to dissect the mechanisms by which gzms influence innate immunity during severe bacterial infection.

## Supplementary Material

Supplementary Data. Supplementary Table 1: Percentage and median fluorescence intensity (MFI) of gzmA and gzmB in diverse lymphocyte populations from WT mice during E. coli peritonitis. Supplementary Table 2: Cell counts of leukocytes, NK cells and granzyme-positive NK cells in peritoneal lavage fluid from wild-type mice during E. coli peritonitis. Supplementary Table 3: Cytokines and chemokine plasma levels of wild-type, gzmA-/-, gzmB-/- and gzmAxB-/- mice during E. coli peritonitis. Supplementary Figure 1: Gating strategy for the analysis of the expression of granzymes A and B by lymphocyte populations in wild-type mice. Intracellular expression of gzmA and B by lymphocyte populations was analysed in peritoneal lavage fluid (PLF) and blood from wild-type mice by flow cytometry. Leukocytes region was gated on the basis of forward (FSC) and side scattering (SSC) characteristics. A. CD8+ T (CD3+CD8+), CD4+ T (CD3+CD4+), γδ T (CD3+γδ TCR+), NK1.1+ T (CD3+NK1.1+) and NK (CD3-NK1.1+) cells were identified by dot-plots, and the percentage of gzm+ cells in each lymphocyte population as well as the median fluorescence intensity (MFI) of the positive expression were determined in histogram plots. B. GzmA+ and gzmB+ cells were identified by histogram plots, and the percentage of cells corresponding to each lymphocyte population within the gzm+ cells were determined in dot-plots. Data shown are of blood from a representative individual (gating of PLF samples was done similarly as for blood). Supplementary Figure 2: Histopathology of liver and lung from wild-type, gzmA-/-, gzmB-/- and gzmAxB-/- mice during E. coli peritonitis. Mice were infected intraperitoneally with 1.3∗104 CFU E. coli and sacrificed at 6, 14 and 20h after infection. Data are box-and-whisker diagrams depicting the smallest observation, lower quartile, median, upper quartile and largest observation. N = 7-8 per group at each time point. ∗ P<0.05, ∗∗ P<0.01 determined by Mann-Whitney U test.

## Figures and Tables

**Table 1:** Plasma levels of ASAT, ALAT, LDH, creatinine and urea, and PLF levels of nucleosomes from wild-type and *gzmA^−/−^*, *gzmB^−/−^*, and *gzmAxB^−/−^* mice at late stage *E. coli* peritonitis (20 h).

	WT	*gzmA^−/−^*	*gzmB^−/−^*	*gzmAxB^−/−^*
ASAT (U/ml)	2.7 (2.3–3.1)	3.3 (0.8–4.4)	1.4 (0.5–5.0)	2.1 (1.4–3.0)
ALAT (U/ml)	0.7 (0.4–0.9)	1.2 (0.3–1.3)	0.6 (0.05–1.3)	0.6 (0.4–0.9)
LDH (U/ml)	3.0 (2.7–4.9)	3.2 (0.5–5.3)	1.6 (0.8–2.9)	1.6 (0.6–4.9)
Creatinine (*μ*mol/l)	6.7 (6.2–7.6)	10.4 (7.7–13.3)^∗^	20.3 (15.9–21.0)^∗∗^^#^	12.1 (8.9–20.0)^∗∗^
Urea (nmol/l)	8.5 (8.1–10.6)	12.6 (8.6–14.4)	14.0 (13.6–18.6)^∗^	11.7 (7.8–13.8)
Nucleosomes (U/ml)	0.8 (0.8–0.8)	10.4 (2.2–21.5)^∗^	6.2 (4.0–9.1)^∗^	8.2 (4.7–10.6)^∗^

Values are median (interquartile range) from 6–8 mice per group 20 h after infection with 1.3∗10^4^ CFU *E. coli*. PLF: peritoneal lavage fluid. ^∗^*P* < 0.05 versus WT, ^∗∗^*P* < 0.01 versus WT, ^#^*P* < 0.05 versus *gzmA^−/−^* by Mann–Whitney *U* test.

## References

[1] Wheeler A. P., Bernard G. R. (1999). Treating patients with severe sepsis. *The New England Journal of Medicine*.

[2] Brook I. (2008). Microbiology and management of abdominal infections. *Digestive Diseases and Science*.

[3] Hecker A., Uhle F., Schwandner T., Padberg W., Weigand M. A. (2014). Diagnostics, therapy and outcome prediction in abdominal sepsis: current standards and future perspectives. *Langenbecks Archives of Surgery*.

[4] Weber G. F., Swirski F. K. (2014). Immunopathogenesis of abdominal sepsis. *Langenbecks Archives of Surgery*.

[5] Susanto O., Trapani J. A., Brasacchio D. (2012). Controversies in granzyme biology. *Tissue Antigens*.

[6] Grossman W. J., Verbsky J. W., Tollefsen B. L., Kemper C., Atkinson J. P., Ley T. J. (2004). Differential expression of granzymes A and B in human cytotoxic lymphocyte subsets and T regulatory cells. *Blood*.

[7] Garcia-Sanz J. A., MacDonald H. R., Jenne D. E., Tschopp J., Nabholz M. (1990). Cell specificity of granzyme gene expression. *The Journal of Immunology*.

[8] Anthony D. A., Andrews D. M., Watt S. V., Trapani J. A., Smyth M. J. (2010). Functional dissection of the granzyme family: cell death and inflammation. *Immunological Reviews*.

[9] Joeckel L. T., Bird P. I. (2014). Are all granzymes cytotoxic in vivo?. *Biological Chemistry*.

[10] Buzza M. S., Bird P. I. (2006). Extracellular granzymes: current perspectives. *Biological Chemistry*.

[11] Zeerleder S., Hack C. E., Caliezi C. (2005). Activated cytotoxic T cells and NK cells in severe sepsis and septic shock and their role in multiple organ dysfunction. *Clinical Immunology*.

[12] Napoli A. M., Fast L. D., Gardiner F., Nevola M., Machan J. T. (2012). Increased granzyme levels in cytotoxic T lymphocytes are associated with disease severity in emergency department patients with severe sepsis. *Shock*.

[13] Lauw F. N., Simpson A. J., Hack C. E. (2000). Soluble granzymes are released during human endotoxemia and in patients with severe infection due to gram-negative bacteria. *The Journal of Infectious Diseases*.

[14] Wensink A. C., Hack C. E., Bovenschen N. (2015). Granzymes regulate proinflammatory cytokine responses. *The Journal of Immunology*.

[15] Metkar S. S., Menaa C., Pardo J. (2008). Human and mouse granzyme A induce a proinflammatory cytokine response. *Immunity*.

[16] Anthony D. A., Andrews D. M., Chow M. (2010). A role for granzyme M in TLR4-driven inflammation and endotoxicosis. *The Journal of Immunology*.

[17] Ebnet K., Hausmann M., Lehmann-Grube F. (1995). Granzyme A-deficient mice retain potent cell-mediated cytotoxicity. *The EMBO Journal*.

[18] Heusel J. W., Wesselschemidt R. L., Chresta S., Russell J. H., Ley T. J. (1994). Cytotoxic lymphocytes require granzyme B for the rapid induction of DNA fragmentation and apoptosis in allogenic target cells. *Cell*.

[19] Simon M. M., Hausmann M., Tran T. (1997). In vitro- and ex vivo-derived cytolytic leukocytes from granzyme A x B double knockout mice are defective in granule-mediated apoptosis but not lysis of target cells. *The Journal of Experimental Medicine*.

[20] Van’t Veer C., van den Pangaart P. S., Kruijswijk D., Florquin S., de Vos A. F., van der Poll T. (2011). Delineation of the role of Toll-like receptor signaling during peritonitis by a gradually growing pathogenic *Escherichia coli*. *The Journal of Biological Chemistry*.

[21] Sewnath M. E., Olszyna D. P., Birjmohun R., ten Kate F. J., Gouma D. J., van Der Poll T. (2001). IL-10 deficient mice demonstrate multiple organ failure and increased mortality during *Escherichia coli* peritonitis despite an accelerated bacterial clearance. *The Journal of Immunology*.

[22] Zeerleder S., Zwart B., te Velthuis H. (2007). A plasma nucleosome releasing factor (NRF) with serine protease activity is instrumental in removal of nucleosomes from secondary necrotic cells. *FEBS Letters*.

[23] Garcia-Laorden M. I., Stroo I., Blok D. C. (2016). Granzymes A and B regulate the local inflammatory response during *Klebsiella pneumoniae* pneumonia. *Journal of Innate Immununity*.

[24] Hagn M., Belz G. T., Kallies A. (2012). Activated mouse B cells lack expression of granzyme B. *The Journal of Immunology*.

[25] Arias M. A., Jiménez de Bagües M. P., Aguiló N. (2014). Elucidating sources and roles of granzymes A and B during bacterial infections and sepsis. *Cell Reports*.

[26] Isailovic N., Daigo K., Mantovani A., Selmi C. (2015). Interleukin-17 and innate immunity in infections and chronic inflammation. *Journal of Autoimmunity*.

[27] Levi M., van der Poll T., Schultz M. (2012). Systemic versus localized coagulation activation contributing to organ failure in critically ill patients. *Seminars in Immunopathology*.

[28] van Lieshout M. H., van der Poll T., van't Veer C. (2014). TLR4 inhibition impairs bacterial clearance in a therapeutic setting in murine abdominal sepsis. *Inflammation Research*.

[29] van Zoelen M. A., Vogl T., Foell D. (2009). Expression and role of myeloid-related protein-14 in clinical and experimental sepsis. *American Journal of Respiratory and Critical Care Medicine*.

[30] Sharron M., Hoptay C. E., Wiles A. A. (2012). Platelets induce apoptosis during sepsis in a contact-dependent manner that is inhibited by GPIIba blockade. *PloS One*.

