# Supplementary material for: Expression and Function of Granzymes A and B in Escherichia coli Peritonitis and Sepsis
Source: Mediators Inflamm. 2017 Jun 12;2017:4137563. doi: 10.1155/2017/4137563 (PMC5485334; doi:10.1155/2017/4137563)
Supplement: Supplementary file 1 — Supplementary Data. Supplementary Table 1: Percentage and median fluorescence intensity (MFI) of gzmA and gzmB in diverse lymphocyte populations from WT mice during E. coli peritonitis. Supplementary Table 2: Cell counts of leukocytes, NK cells and granzyme-positive NK cells in peritoneal lavage fluid from wild-type mice during E. coli peritonitis. Supplementary Table 3: Cytokines and chemokine plasma levels of wild-type, gzmA-/-, gzmB-/- and gzmAxB-/- mice during E. coli peritonitis. Supplementary Figure 1: Gating strategy for the analysis of the expression of granzymes A and B by lymphocyte populations in wild-type mice. Intracellular expression of gzmA and B by lymphocyte populations was analysed in peritoneal lavage fluid (PLF) and blood from wild-type mice by flow cytometry. Leukocytes region was gated on the basis of forward (FSC) and side scattering (SSC) characteristics. A. CD8+ T (CD3+CD8+), CD4+ T (CD3+CD4+), γδ T (CD3+γδ TCR+), NK1.1+ T (CD3+NK1.1+) and NK (CD3-NK1.1+) cells were identified by dot-plots, and the percentage of gzm+ cells in each lymphocyte population as well as the median fluorescence intensity (MFI) of the positive expression were determined in histogram plots. B. GzmA+ and gzmB+ cells were identified by histogram plots, and the percentage of cells corresponding to each lymphocyte population within the gzm+ cells were determined in dot-plots. Data shown are of blood from a representative individual (gating of PLF samples was done similarly as for blood). Supplementary Figure 2: Histopathology of liver and lung from wild-type, gzmA-/-, gzmB-/- and gzmAxB-/- mice during E. coli peritonitis. Mice were infected intraperitoneally with 1.3∗104 CFU E. coli and sacrificed at 6, 14 and 20h after infection. Data are box-and-whisker diagrams depicting the smallest observation, lower quartile, median, upper quartile and largest observation. N = 7-8 per group at each time point. ∗ P<0.05, ∗∗ P<0.01 determined by Mann-Whitney U test. [file 4137563.f1.docx]

SUPPLEMENTARY DATA

Expression and Function of Granzymes A and B in *Escherichia coli* Peritonitis and Sepsis

M. Isabel García-Laorden^1^, Ingrid Stroo^1,2^, Sanne Terpstra^1^, Sandrine Florquin^3^, Jan Paul Medema^1,4^, Cornelis van´t Veer^1^, Alex F. de Vos^1^, Tom van der Poll^1,5^.

^1^Center for Experimental and Molecular Medicine (CEMM), Academic Medical Center/ University of Amsterdam, Meibergdreef 9, 1105 AZ, Amsterdam, The Netherlands.

^2^Department of Immunopathology, Sanquin Research, Plesmanlaan 125, 1066 CX, Amsterdam, The Netherlands.

^3^Department of Pathology, Academic Medical Center/ University of Amsterdam, Meibergdreef 9, 1105 AZ, Amsterdam, The Netherlands.

^4^Laboratory for Experimental Oncology and Radiobiology (LEXOR), Academic Medical Center, Meibergdreef 9, 1105 AZ, Amsterdam, The Netherlands.

^5^Division of Infectious Diseases. Academic Medical Center/ University of Amsterdam, Meibergdreef 9, 1105 AZ, Amsterdam, The Netherlands.

Correspondence should be addressed to: M. Isabel García-Laorden, Academic Medical Center, Meibergdreef 9, Room G2-130, 1105 AZ Amsterdam, The Netherlands. Phone: +31-20-5665910; Fax: + 31-206977192; E-mail: ihalemgl@yahoo.es

**Supplementary Table 1:** Percentage and median fluorescence intensity (MFI) of gzmA and gzmB in diverse lymphocyte populations from WT mice during *E. coli* peritonitis.

| **A** | Granzyme A | | | | | | | | Granzyme B | | | | | | | |
| --- | --- | --- | --- | --- | --- | --- | --- | --- | --- | --- | --- | --- | --- | --- | --- | --- |
|  | CD8+T | | CD4+T | | γδ T | | NK1.1+T | | CD8+T | | CD4+T | | γδ T | | NK1.1+T | |
| 0h % | 1.43 | | 0.18 | | 1.43 | | 3.97 | | 1.07 | | 0.12 | | 0.44 | | 3.03 | |
| MFI | 384.00 | | 408.00 | | 375.00 | | 522.50 | | 549.00 | | 429.00 | | 404.50 | | 595.00 | |
| 6h % | 5.38 | | 0.55 | | 2.62 | | 7.67 | | 3.88 | | 1.27 | | 1.45 | | 6.76 | |
| MFI | 420.50 | | 380.00 | | 421.50 | | 1125.00 | | 653.00 | | 545.00 | | 496.00 | | 815.00 | |
| 14h % | 9.42 | | 2.55 | | 2.47 | | 25.00 | | 13.55 | | 3.44 | | 8.64 | | 34.25 | |
| MFI | 353.50 | | 344.00 | | 524.00 | | 997.00 | | 497.50 | | 485.00 | | 614.00 | | 3173.00 | |
| 20h % | 28.70 | | 9.60 | | 39.96 | | 45.15 | | 6.38 | | 7.09 | | 14.20 | | 18.30 | |
| MFI | 436.00 | | 470.00 | | 1080.50 | | 780.00 | | 631.00 | | 404.00 | | 580.50 | | 667.50 | |
|  | % | MFI | % | MFI | % | MFI | % | MFI | % | MFI | % | MFI | % | MFI | % | MFI |
| P 0 vs 6 | * | ns | * | ns | ns | ns | ** | * | ** | ns | ** | ns | * | ns | * | ns |
| P 0 vs 14 | ** | ns | ** | ns | ns | ns | ** | ** | ** | ns | ** | ns | ns | ns | ** | ** |
| P 0 vs 20 | ** | ns | ** | ns | ** | * | ** | ns | ** | ns | ** | ns | ** | ns | ns | ns |
| P 6 vs 14 | ns | ns | ** | ns | ns | ns | ** | ns | * | ns | * | ns | ns | ns | ** | ** |
| P 6 vs 20 | ** | ns | ** | ns | * | ns | * | ns | ns | ns | ns | ns | ns | * | ns | ns |
| P14 vs 20 | ** | ns | * | ns | ns | ns | ns | ns | ns | ns | ns | ns | ns | ns | ns | ** |

| **B** | Granzyme A | | | | | | | | Granzyme B | | | | | | | |
| --- | --- | --- | --- | --- | --- | --- | --- | --- | --- | --- | --- | --- | --- | --- | --- | --- |
|  | CD8+T | | CD4+T | | γδ T | | NK1.1+T | | CD8+T | | CD4+T | | γδ T | | NK1.1+T | |
| 0h % | 0.34 | | 0.26 | | 1.14 | | 18.80 | | 0.05 | | 0.04 | | 0.24 | | 6.57 | |
| MFI | 760.00 | | 879.00 | | 684.00 | | 1078.00 | | 786.00 | | 764.00 | | 1445.00 | | 653.00 | |
| 6h % | 1.16 | | 0.39 | | 2.75 | | 19.60 | | 1.08 | | 0.43 | | 3.16 | | 21.10 | |
| MFI | 470.00 | | 528.50 | | 398.50 | | 1049.50 | | 569.50 | | 544.50 | | 472.50 | | 755.00 | |
| 14h % | 1.68 | | 0.42 | | 6.85 | | 44.00 | | 2.34 | | 0.80 | | 6.64 | | 51.55 | |
| MFI | 470.00 | | 566.00 | | 870.50 | | 1438.00 | | 549.00 | | 592.00 | | 696.00 | | 1786.50 | |
| 20h % | 1.76 | | 1.15 | | 3.36 | | 36.30 | | 2.13 | | 1.04 | | 4.03 | | 36.75 | |
| MFI | 510.00 | | 475.00 | | 407.00 | | 1133.00 | | 513.50 | | 458.00 | | 434.00 | | 1090.50 | |
|  | % | MFI | % | MFI | % | MFI | % | MFI | % | MFI | % | MFI | % | MFI | % | MFI |
| P 0 vs 6 | * | * | ns | ** | ns | * | ns | ns | * | ns | ** | * | ns | * | ns | ns |
| P 0 vs 14 | ** | * | ns | * | ** | ns | ** | ns | ** | ns | ** | * | ** | ns | ** | * |
| P 0 vs 20 | ns | * | ** | * | * | * | ns | ns | * | ns | * | ** | ns | * | ns | ns |
| P 6 vs 14 | ns | ns | ns | ns | * | * | ** | * | * | ns | ns | ns | ns | ns | ** | ** |
| P 6 vs 20 | ns | ns | * | ns | ns | ns | ns | ns | ns | ns | ns | * | ns | ns | ns | ns |
| P14 vs 20 | ns | ns | ns | ns | ns | * | ns | ns | ns | ns | ** | ns | ** | ns | ns | ns |

Values are medians from 5-6 mice of the percentage of each population of lymphocytes expressing gzmA or gzmB and the MFI in these cells, in uninfected mice and 6, 14 and 20h after infection with 1.3*10^4^ CFU *E. coli*. A. Values in lymphocyte populations from PLF. B. Values in lymphocyte populations from blood. P values of the comparisons between time points in each lymphocyte population were determined by Mann-Whitney U test. ns: non-significant , * P<0.05, ** P<0.01.

**Supplementary Table 2:** Cell counts of leukocytes, NK cells and granzyme-positive NK cells in peritoneal lavage fluid from wild-type mice during *E. coli* peritonitis.

|  | 0h | 6h | 14h | 20h |
| --- | --- | --- | --- | --- |
| Leukocytes (cells/ml) | 5.5*10^5^  [5.1*10^5^-6.72*10^5^] | 2.1*10^5 *^  [1.7*10^5^-3.8*10^5^] | 1.1*10^6 * **^  [1.0*10^6^-1.2*10^6^] | 2.8*10^6 * **^  [1.3*10^6^-4.2*10^6^] |
| NK (%) | 2.18  [2.1-2.3] | 4.1 ^*^  [3.8-4.3] | 5.1  [4.4-5.8] | 14.7 ^* **^  [6.1-26.6] |
| NK (cells/ml) | 1.3*10^4^  [1.1*10^4^-1.4*10^4^] | 1.0*10^4 *^  [7.7*10^3^-1.3*10^4^] | 4.8*10^4 **^  [4.7*10^4^-6.8*10^4^] | 2.7*10^5 * ** #^  [1.8*10^5^-3.4*10^5^] |
| gzmA^+^ NK cells (cells/ml) | 2.7*10^3^  [2.6*10^3^-3.5*10^3^] | 1.4*10^3 *^  [1.1*10^3^-2.0*10^3^] | 3.1*10^3^  [2.1*10^3^-3.8*10^3^] | 3.7*10^3 **^  [3.2*10^3^-5.1*10^3^] |
| gzmB^+^ NK cells (cells/ml) | 5.5*10^2^  [4.0*10^2^-7.2*10^2^] | 1.2*10^3^  [5.2*10^2^-2.0*10^3^] | 3.6*10^3 * ##^  [2.6*10^3^-5.7*10^3^] | 2.8*10^3 §^  [1.2*10^3^-3.7*10^3^] |

Values are medians (interquartile range) from 6 mice per group in uninfected mice and 6, 14 and 20h after infection with 1.3*10^4^ CFU *E. coli*. P values of the comparisons between time points were determined by Mann-Whitney U test. ^*^P<0.01 *vs* 0h, ^**^P<0.01 *vs* 6h, ^#^P<0.01 *vs* 14h, ^##^P<0.05 *vs* 6h, ^§^P<0.05 *vs* 0h.

**Supplementary Table 3:** Cytokines and chemokine plasma levels of wild-type, *gzmA^-/-^*, *gzmB^-/-^* and *gzmAxB^-/-^* mice during *E. coli* peritonitis.

|  | TNF-α (pg/ml) | IFN-γ (pg/ml) | IL-10 (pg/ml) | MCP-1 (pg/ml) |
| --- | --- | --- | --- | --- |
| 6h |  |  |  |  |
| WT | 333.1  [65.0-449.8] | 2.1  [2.0-2.8] | 5.0  [0.0-5.0] | 461.4  [211.2-2478.1] |
| *gzmA^-/-^* | 380.4  [246.8-1154.5] | 1.9  [1.8-2.4] | 5.0  [5.0-10.2] | 2402.4  [1784.3-8856.3] |
| *gzmB^-/-^* | 509.1  [277.9-567.6] | 1.6  [1.3-3.7] | 11.1  [5.0-15.0] | 4944.6^*^  [2913.9-10000.0] |
| *gzmAxB^-/-^* | 352.6  [160.7-722.3] | 2.1  [1.7-3.3] | 5.0  [0.0-20.6] | 3109.9  [1673.3-7177.0] |
| 14h |  |  |  |  |
| WT | 242.9  [191.1-658.5] | 13.9  [8.7-18.7] | 26.0  [10.3-39.3] | 3226.9  [2048.5-9119.0] |
| *gzmA^-/-^* | 504.3  [345.1-905.8] | 4.9  [3.7-8.2] | 42.8  [35.4-55.3] | 4794.1  [3412.8-9057.4] |
| *gzmB^-/-^* | 340.0  [211.8-403.5] | 3.4  [3.0-5.5] | 39.2  [27.2-87.2] | 4194.4  [2537.9-5862.2] |
| *gzmAxB^-/-^* | 500.5  [343.9-594.6] | 3.7^A^  [3.0-4.2] | 55.0^A^  [48.2-60.3] | 6612.6  [3768.6-8089.8] |
| 20h |  |  |  |  |
| WT | 359.8  [306.0-379.1] | 2.9  [2.2-3.6] | 30.1  [21.8-38.7] | 5106.1  [3644.4-5946.9] |
| *gzmA^-/-^* | 471.0  [206.6-595.7] | 2.1  [0.0-5.0] | 38.7  [13.3-94.1] | 5759.5  [2819.2-7402.9] |
| *gzmB^-/-^* | 460.7  [423.1-683.0] | 11.7  [8.9-30.6] | 47.2  [36.6-49.8] | 6246.2  [5911.7-6921.4] |
| *gzmAxB^-/-^* | 305.0  [263.6-737.4] | 0.0^#^  [0.0-2.2] | 34.5  [24.3-74.9] | 4724.6  [3326.6-5834.8] |

Values are medians (interquartile range) from 7-8 mice per group 6, 14 and 20h after infection with 1.3*10^4^ CFU *E. coli*. ^*^P<0.05 *vs* WT, ^#^P<0.05 *vs* *gzmB^-/-^* by Mann-Whitney U test.


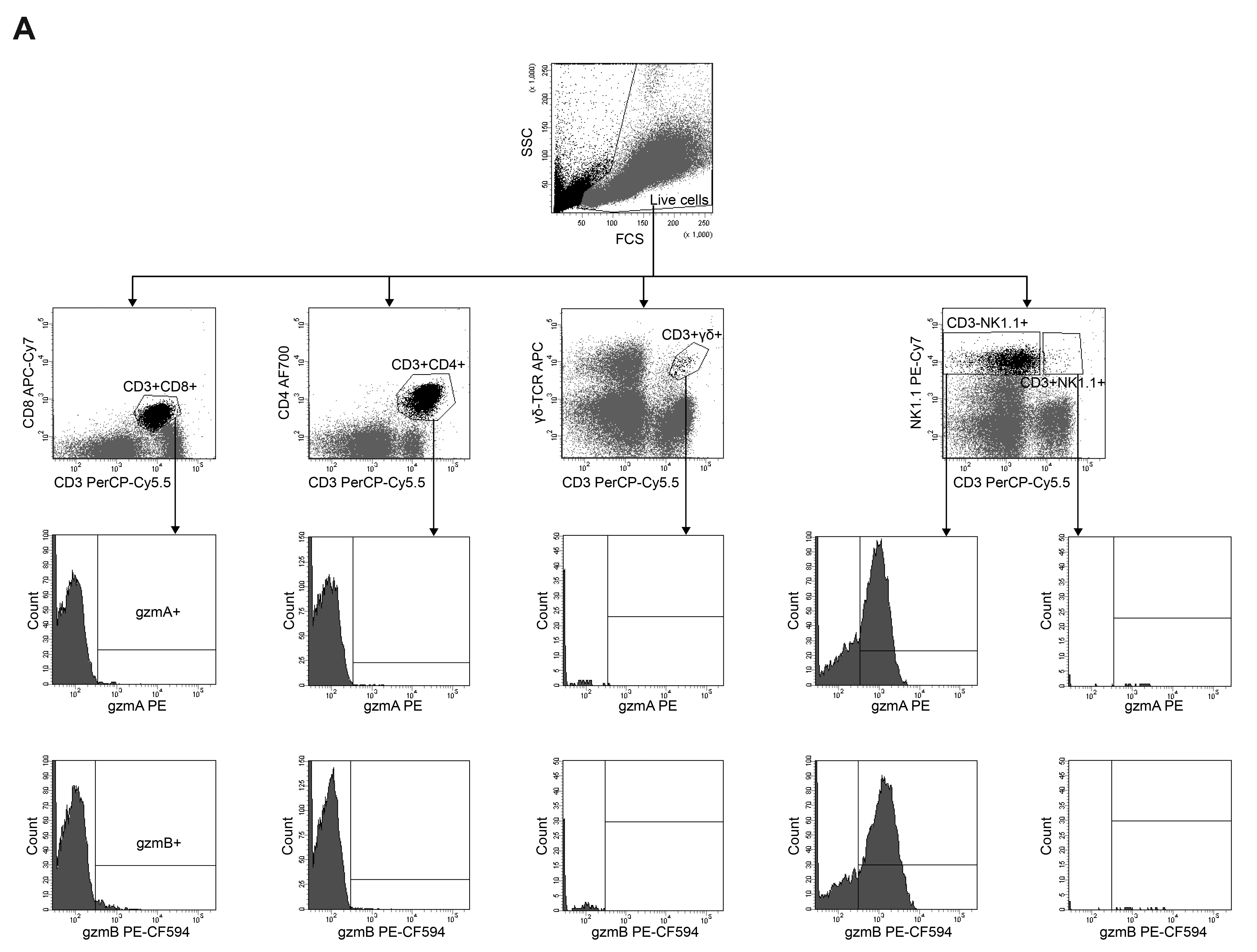


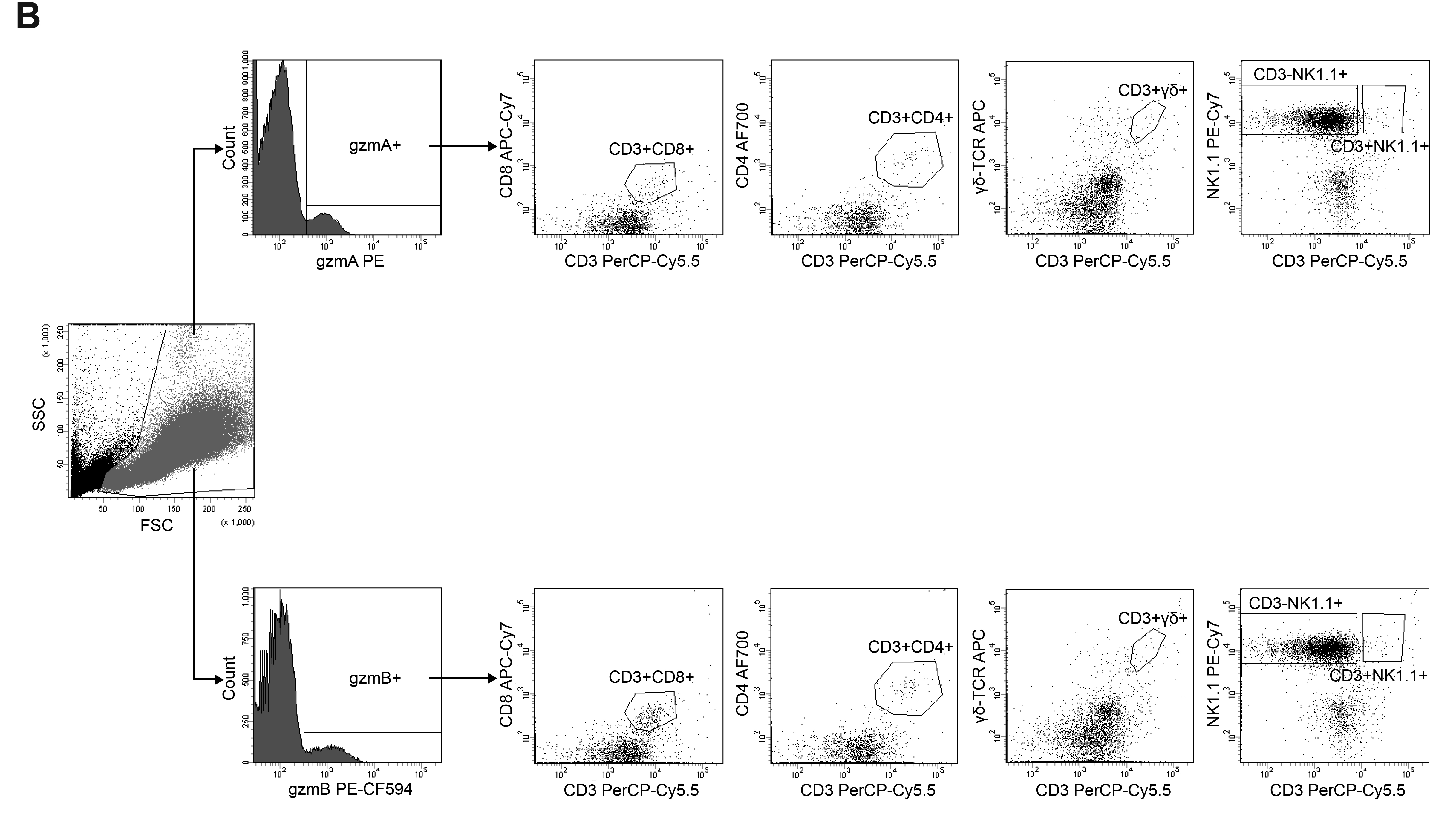


**Supplementary Figure 1: Gating strategy for the analysis of the expression of granzymes A and B by lymphocyte populations in wild-type mice.** Intracellular expression of gzmA and B by lymphocyte populations was analysed in peritoneal lavage fluid (PLF) and blood from wild-type mice by flow cytometry. Leukocytes region was gated on the basis of forward (FSC) and side scattering (SSC) characteristics. A. CD8^+^ T (CD3^+^CD8^+^), CD4^+^ T (CD3^+^CD4^+^), ɣδ T (CD3^+^ɣδ TCR^+^), NK1.1^+^ T (CD3^+^NK1.1^+^) and NK (CD3^-^NK1.1^+^) cells were identified by dot-plots, and the percentage of gzm^+^ cells in each lymphocyte population as well as the median fluorescence intensity (MFI) of the positive expression were determined in histogram plots. B. GzmA^+^ and gzmB^+^ cells were identified by histogram plots, and the percentage of cells corresponding to each lymphocyte population within the gzm^+^ cells were determined in dot-plots. Data shown are of blood from a representative individual (gating of PLF samples was done similarly as for blood).


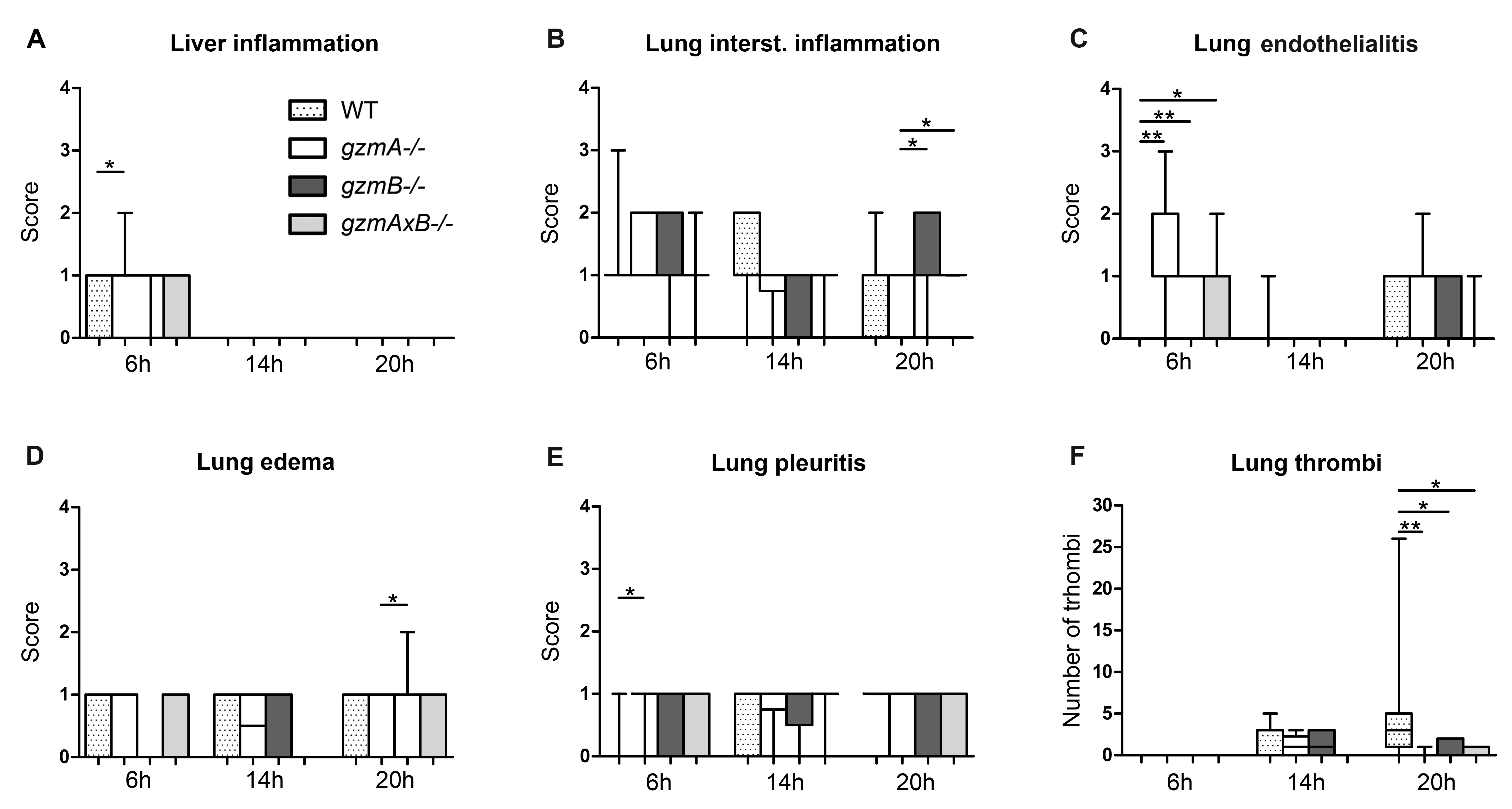


**Supplementary Figure 2: Histopathology of liver and lung from wild-type, *gzmA^-/-^*, *gzmB^-/-^* and *gzmAxB^-/-^* mice during *E. coli* peritonitis.** Mice were infected intraperitoneally with 1.3*10^4^ CFU *E. coli* and sacrificed at 6, 14 and 20h after infection. Data are box-and-whisker diagrams depicting the smallest observation, lower quartile, median, upper quartile and largest observation. N = 7-8 per group at each time point. * P<0.05, ** P<0.01 determined by Mann-Whitney U test.
