# Supplementary material for: Expression and Function of Granzymes A and B in Escherichia coli Peritonitis and Sepsis
Source: Mediators Inflamm. 2017 Jun 12;2017:4137563. doi: 10.1155/2017/4137563 (PMC5485334; doi:10.1155/2017/4137563)
Supplement: Supplementary file 2 [file 4137563.f2.pdf]

SUPPLEMENTARY DATA

Expression and Function of Granzymes A and B in *Escherichia coli* Peritonitis and Sepsis

M. Isabel García-Laorden[1], Ingrid Stroo[1,2], Sanne Terpstra[1], Sandrine Florquin[3], Jan Paul Medema[1,4], Cornelis van´t Veer[1], Alex F. de Vos[1], Tom van der Poll[1,5].

[1]Center for Experimental and Molecular Medicine (CEMM), Academic Medical Center/ University of Amsterdam, Meibergdreef 9, 1105 AZ, Amsterdam, The Netherlands.

[2]Department of Immunopathology, Sanquin Research, Plesmanlaan 125, 1066 CX, Amsterdam, The Netherlands.

[3]Department of Pathology, Academic Medical Center/ University of Amsterdam, Meibergdreef 9, 1105 AZ, Amsterdam, The Netherlands.

[4]Laboratory for Experimental Oncology and Radiobiology (LEXOR), Academic Medical Center, Meibergdreef 9, 1105 AZ, Amsterdam, The Netherlands.

[5]Division of Infectious Diseases. Academic Medical Center/ University of Amsterdam, Meibergdreef 9, 1105 AZ, Amsterdam, The Netherlands.

Correspondence should be addressed to: M. Isabel García-Laorden, Academic Medical Center, Meibergdreef 9, Room G2-130, 1105 AZ Amsterdam, The Netherlands. Phone: +31-20-5665910; Fax: + 31-206977192; E-mail: ihalemgl@yahoo.es

Supplementary Table 1: Percentage and median fluorescence intensity (MFI) of gzmA and gzmB in diverse lymphocyte populations from WT mice during *E. coli* peritonitis.

A

		Granzyme A				Granzyme B			
		CD8+T	CD4+T	γδ T	NK1.1+T	CD8+T	CD4+T	γδ T	NK1.1+T
0h	%	1.43	0.18	1.43	3.97	1.07	0.12	0.44	3.03
	MFI	384.00	408.00	375.00	522.50	549.00	429.00	404.50	595.00
6h	%	5.38	0.55	2.62	7.67	3.88	1.27	1.45	6.76
	MFI	420.50	380.00	421.50	1125.00	653.00	545.00	496.00	815.00
14h	%	9.42	2.55	2.47	25.00	13.55	3.44	8.64	34.25
	MFI	353.50	344.00	524.00	997.00	497.50	485.00	614.00	3173.00
20h	%	28.70	9.60	39.96	45.15	6.38	7.09	14.20	18.30
	MFI	436.00	470.00	1080.50	780.00	631.00	404.00	580.50	667.50

	%	MFI	%	MFI	%	MFI	%	MFI	%	MFI	%	MFI	%	MFI	%	MFI
P 0 vs 6	*	ns	*	ns	ns	ns	**	*	**	ns	**	ns	*	ns	*	ns
P 0 vs 14	**	ns	**	ns	ns	ns	**	**	**	ns	**	ns	ns	ns	**	**
P 0 vs 20	**	ns	**	ns	**	*	**	ns	**	ns	**	ns	**	ns	ns	ns
P 6 vs 14	ns	ns	**	ns	ns	ns	**	ns	*	ns	*	ns	ns	ns	**	**
P 6 vs 20	**	ns	**	ns	*	ns	*	ns	ns	ns	ns	ns	ns	*	ns	ns
P14 vs 20	**	ns	*	ns	ns	ns	ns	ns	ns	ns	ns	ns	ns	ns	ns	**

B

		Granzyme A				Granzyme B			
		CD8+T	CD4+T	γδ T	NK1.1+T	CD8+T	CD4+T	γδ T	NK1.1+T
0h	%	0.34	0.26	1.14	18.80	0.05	0.04	0.24	6.57
	MFI	760.00	879.00	684.00	1078.00	786.00	764.00	1445.00	653.00
6h	%	1.16	0.39	2.75	19.60	1.08	0.43	3.16	21.10
	MFI	470.00	528.50	398.50	1049.50	569.50	544.50	472.50	755.00
14h	%	1.68	0.42	6.85	44.00	2.34	0.80	6.64	51.55
	MFI	470.00	566.00	870.50	1438.00	549.00	592.00	696.00	1786.50
20h	%	1.76	1.15	3.36	36.30	2.13	1.04	4.03	36.75
	MFI	510.00	475.00	407.00	1133.00	513.50	458.00	434.00	1090.50

	%	MFI	%	MFI	%	MFI	%	MFI	%	MFI	%	MFI	%	MFI	%	MFI
P 0 vs 6	*	*	ns	**	ns	*	ns	ns	*	ns	**	*	ns	*	ns	ns
P 0 vs 14	**	*	ns	*	**	ns	**	ns	**	ns	**	*	**	ns	**	*
P 0 vs 20	ns	*	**	*	*	*	ns	ns	*	ns	*	**	ns	*	ns	ns
P 6 vs 14	ns	ns	ns	ns	*	*	**	*	*	ns	ns	ns	ns	ns	**	**
P 6 vs 20	ns	ns	*	ns	ns	ns	ns	ns	ns	ns	ns	*	ns	ns	ns	ns
P14 vs 20	ns	ns	ns	ns	ns	*	ns	ns	ns	ns	**	ns	**	ns	ns	ns

Values are medians from 5-6 mice of the percentage of each population of lymphocytes expressing gzmA or gzmB and the MFI in these cells, in uninfected mice and 6, 14 and 20h after infection with $1.3*10^4$ CFU *E. coli*. A. Values in lymphocyte populations from PLF. B. Values in lymphocyte populations from blood. P values of the comparisons between time points in each lymphocyte population were determined by Mann-Whitney U test. ns: non-significant , * P<0.05, ** P<0.01.

Supplementary Table 2: Cell counts of leukocytes, NK cells and granzyme-positive NK cells in peritoneal lavage fluid from wild-type mice during *E. coli* peritonitis.

	0h	6h	14h	20h
Leukocytes (cells/ml)	$5.5*10^5$ [$5.1*10^5$-$6.72*10^5$]	$2.1*10^{5\,*}$ [$1.7*10^5$-$3.8*10^5$]	$1.1*10^{6\,*\,**}$ [$1.0*10^6$-$1.2*10^6$]	$2.8*10^{6\,*\,**}$ [$1.3*10^6$-$4.2*10^6$]
NK (%)	2.18 [2.1-2.3]	4.1^{*} [3.8-4.3]	5.1 [4.4-5.8]	$14.7^{*\,**}$ [6.1-26.6]
NK (cells/ml)	$1.3*10^4$ [$1.1*10^4$-$1.4*10^4$]	$1.0*10^{4\,*}$ [$7.7*10^3$-$1.3*10^4$]	$4.8*10^{4\,**}$ [$4.7*10^4$-$6.8*10^4$]	$2.7*10^{5\,*\,**\,\#}$ [$1.8*10^5$-$3.4*10^5$]
gzmA$^+$ NK cells (cells/ml)	$2.7*10^3$ [$2.6*10^3$-$3.5*10^3$]	$1.4*10^{3\,*}$ [$1.1*10^3$-$2.0*10^3$]	$3.1*10^3$ [$2.1*10^3$-$3.8*10^3$]	$3.7*10^{3\,**}$ [$3.2*10^3$-$5.1*10^3$]
gzmB$^+$ NK cells (cells/ml)	$5.5*10^2$ [$4.0*10^2$-$7.2*10^2$]	$1.2*10^3$ [$5.2*10^2$-$2.0*10^3$]	$3.6*10^{3\,*\,\#\#}$ [$2.6*10^3$-$5.7*10^3$]	$2.8*10^{3\,\S}$ [$1.2*10^3$-$3.7*10^3$]

Values are medians (interquartile range) from 6 mice per group in uninfected mice and 6, 14 and 20h after infection with $1.3*10^4$ CFU *E. coli*. P values of the comparisons between time points were determined by Mann-Whitney U test. *P<0.01 *vs* 0h, **P<0.01 *vs* 6h, $^{\#}$P<0.01 *vs* 14h, $^{\#\#}$P<0.05 *vs* 6h, §P<0.05 *vs* 0h.

Supplementary Table 3: Cytokines and chemokine plasma levels of wild-type, *gzmA*$^{-/-}$, *gzmB*$^{-/-}$ and *gzmAxB*$^{-/-}$ mice during *E. coli* peritonitis.

	TNF-α (pg/ml)	IFN-γ (pg/ml)	IL-10 (pg/ml)	MCP-1 (pg/ml)
**6h**				
WT	333.1 [65.0-449.8]	2.1 [2.0-2.8]	5.0 [0.0-5.0]	461.4 [211.2-2478.1]
gzmA$^{-/-}$	380.4 [246.8-1154.5]	1.9 [1.8-2.4]	5.0 [5.0-10.2]	2402.4 [1784.3-8856.3]
gzmB$^{-/-}$	509.1 [277.9-567.6]	1.6 [1.3-3.7]	11.1 [5.0-15.0]	4944.6[*] [2913.9-10000.0]
gzmAxB$^{-/-}$	352.6 [160.7-722.3]	2.1 [1.7-3.3]	5.0 [0.0-20.6]	3109.9 [1673.3-7177.0]
**14h**				
WT	242.9 [191.1-658.5]	13.9 [8.7-18.7]	26.0 [10.3-39.3]	3226.9 [2048.5-9119.0]
gzmA$^{-/-}$	504.3 [345.1-905.8]	4.9 [3.7-8.2]	42.8 [35.4-55.3]	4794.1 [3412.8-9057.4]
gzmB$^{-/-}$	340.0 [211.8-403.5]	3.4 [3.0-5.5]	39.2 [27.2-87.2]	4194.4 [2537.9-5862.2]
gzmAxB$^{-/-}$	500.5 [343.9-594.6]	3.7[A] [3.0-4.2]	55.0[A] [48.2-60.3]	6612.6 [3768.6-8089.8]
**20h**				
WT	359.8 [306.0-379.1]	2.9 [2.2-3.6]	30.1 [21.8-38.7]	5106.1 [3644.4-5946.9]
gzmA$^{-/-}$	471.0 [206.6-595.7]	2.1 [0.0-5.0]	38.7 [13.3-94.1]	5759.5 [2819.2-7402.9]
gzmB$^{-/-}$	460.7 [423.1-683.0]	11.7 [8.9-30.6]	47.2 [36.6-49.8]	6246.2 [5911.7-6921.4]
gzmAxB$^{-/-}$	305.0 [263.6-737.4]	0.0[#] [0.0-2.2]	34.5 [24.3-74.9]	4724.6 [3326.6-5834.8]

Values are medians (interquartile range) from 7-8 mice per group 6, 14 and 20h after infection with $1.3*10^4$ CFU *E. coli*. [*]P<0.05 *vs* WT, [#]P<0.05 *vs* *gzmB*$^{-/-}$ by Mann-Whitney U test.

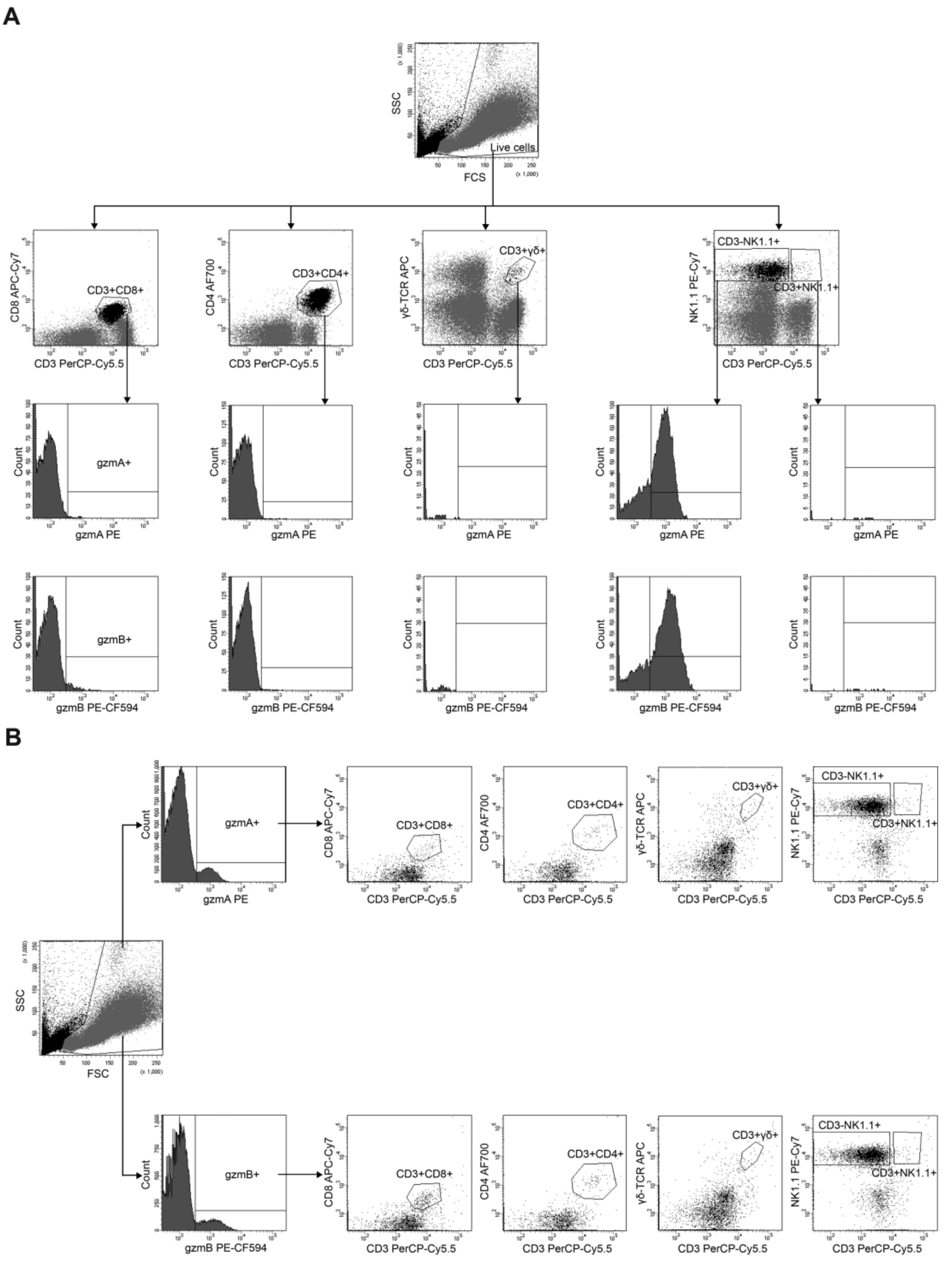

Supplementary Figure 1: Gating strategy for the analysis of the expression of granzymes A and B by lymphocyte populations in wild-type mice. Intracellular expression of gzmA and B by lymphocyte populations was analysed in peritoneal lavage fluid (PLF) and blood from wild-type mice by flow cytometry. Leukocytes region was gated on the basis of forward (FSC) and side scattering (SSC) characteristics. A. CD8$^+$ T (CD3$^+$CD8$^+$), CD4$^+$ T (CD3$^+$CD4$^+$), γδ T (CD3$^+$γδ TCR$^+$), NK1.1$^+$ T (CD3$^+$NK1.1$^+$) and NK (CD3$^-$NK1.1$^+$) cells were identified by dot-plots, and the percentage of gzm$^+$ cells in each lymphocyte population as well as the median fluorescence intensity (MFI) of the positive expression were determined in histogram plots. B. GzmA$^+$ and

$gzmB^+$ cells were identified by histogram plots, and the percentage of cells corresponding to each lymphocyte population within the gzm^+ cells were determined in dot-plots. Data shown are of blood from a representative individual (gating of PLF samples was done similarly as for blood).

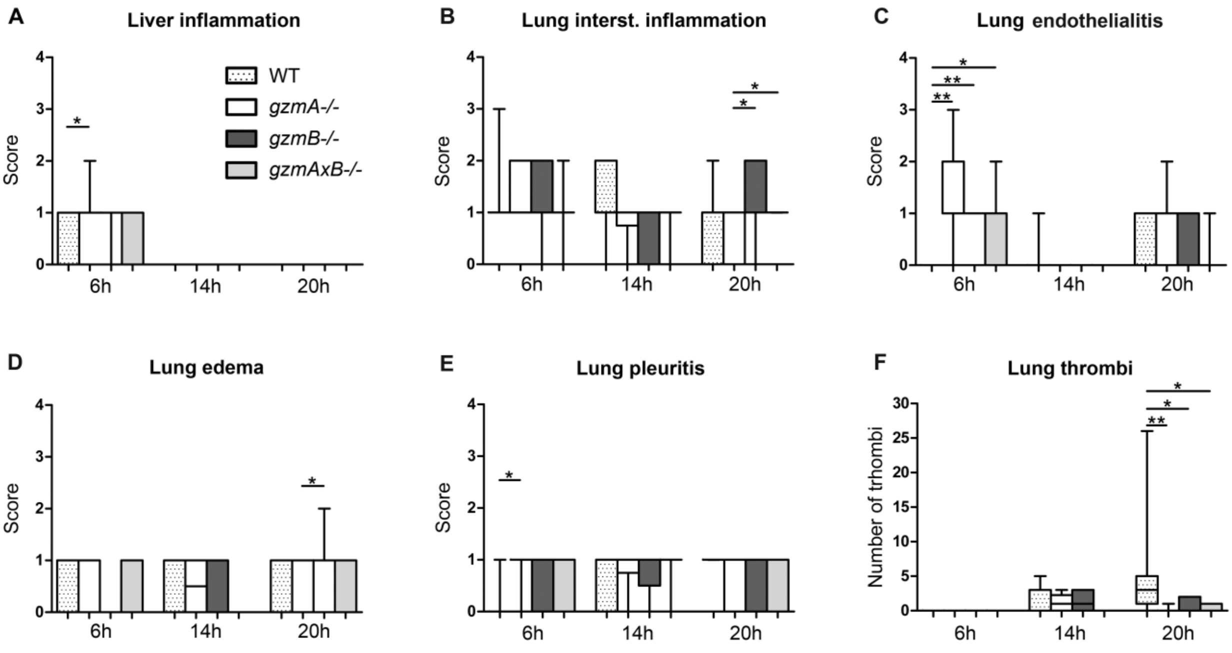

Supplementary Figure 2: Histopathology of liver and lung from wild-type, *gzmA*$^{-/-}$, *gzmB*$^{-/-}$ and *gzmAxB*$^{-/-}$ mice during *E. coli* peritonitis. Mice were infected intraperitoneally with 1.3*10^4 CFU *E. coli* and sacrificed at 6, 14 and 20h after infection. Data are box-and-whisker diagrams depicting the smallest observation, lower quartile, median, upper quartile and largest observation. N = 7-8 per group at each time point. * P<0.05, ** P<0.01 determined by Mann-Whitney U test.
