# Supplementary figures and images for: Expression and Function of Granzymes A and B in Escherichia coli Peritonitis and Sepsis
Source: Mediators Inflamm. 2017 Jun 12;2017:4137563. doi: 10.1155/2017/4137563 (PMC5485334; doi:10.1155/2017/4137563)

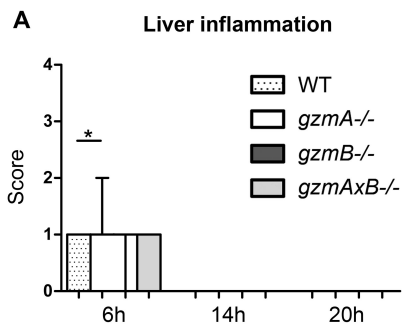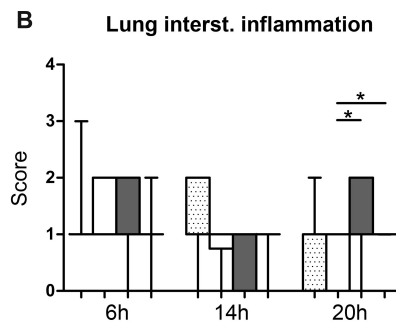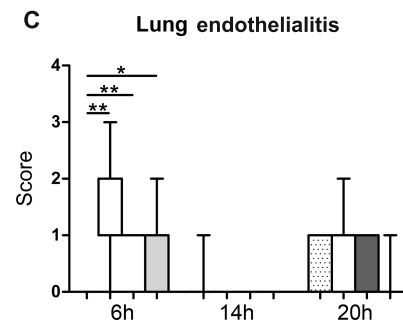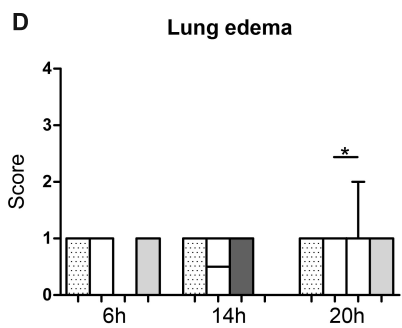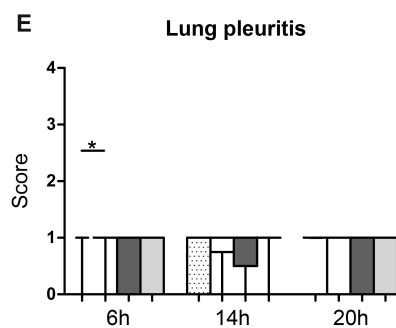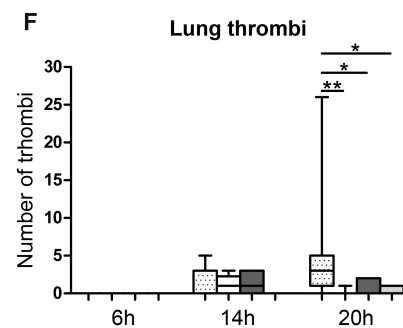

Supplement: Supplementary file 4 [file 4137563.f4.pdf]
